# The Protective Effect of Thiamine and Thiamine Pyrophosphate Against Linezolid-Induced Oxidative Liver Damage and Lactic Acidosis in Rats

**DOI:** 10.3390/antiox14080920

**Published:** 2025-07-27

**Authors:** Bahar Isik, Irem Ates, Nurinisa Yucel, Bahadir Suleyman, Ali Sefa Mendil, Esra Tuba Sezgin, Halis Suleyman

**Affiliations:** 1 Department of Emergency Medicine, Faculty of Medicine, Erzincan Binali Yildirim University, Erzincan 24100, Turkey; bahar.isik@erzincan.edu.tr; 2 Department of Anesthesiology and Reanimation, Faculty of Medicine, Atatürk University, Erzurum 25240, Turkey; driremates@hotmail.com; 3 Vocational School of Health Services, Pharmacy Services Program, Erzincan Binali Yildirim University, Erzincan 24100, Turkey; nurinisa.yucel@erzincan.edu.tr; 4 Department of Pharmacology, Faculty of Medicine, Erzincan Binali Yildirim University, Erzincan 24100, Turkey; bsuleyman@erzincan.edu.tr; 5 Department of Pathology, Faculty of Veterinary Medicine, Erciyes University, Kayseri 38280, Turkey; sefaali5252@gmail.com; 6 Vocational School of Health Services, Anesthesia Program, Erzincan Binali Yildirim University, Erzincan 24100, Turkey; esra.demir@erzincan.edu.tr

**Keywords:** linezolid, thiamine pyrophosphate, liver, oxidative stress, lactic acidosis

## Abstract

Linezolid, an antimicrobial agent, has been linked to lactic acidosis, oxidative stress, and liver damage. Oxidative stress is considered to play a key role in this damage. Thiamine pyrophosphate (TPP), the active form of thiamine, may prevent lactate accumulation and enhance aerobic capacity. Therefore, this study aimed to evaluate the protective effect of TPP against possible linezolid-induced liver damage and lactic acidosis in rats. Twenty-four male Wistar albino rats were randomly assigned to four groups (*n* = 6): healthy control (HG), linezolid (LZD), thiamine plus linezolid (TLZD), and TPP plus linezolid (TPLZD). Thiamine and TPP (20 mg/kg, intraperitoneal (i.p.)) were administered once daily, while linezolid (125 mg/kg, per os (p.o.)) was given twice daily (250 mg/kg/day) for 28 days. Animals were euthanized under high-dose anesthesia (with 50 mg/kg, i.p. thiopental sodium). Liver tissues were analyzed for MDA, tGSH, SOD, and CAT, and examined histopathologically. Blood samples were collected prior to euthanasia to assess lactate, LDH, ALT, AST, and TPP levels. In the LZD group, MDA, lactate, ALT, AST, and LDH levels significantly increased, while tGSH, SOD, CAT, and TPP decreased (*p* < 0.001). Histopathology showed hydropic degeneration, necrosis, and mononuclear cell infiltration (*p* < 0.05). Thiamine did not prevent these alterations (*p* > 0.05), whereas TPP significantly prevented both biochemical and histopathological changes (*p* < 0.05), indicating its protective efficacy. TPP may offer significant protection against linezolid-induced hepatotoxicity and lactic acidosis.

## 1. Introduction

Linezolid is a synthetic antimicrobial agent classified as an oxazolidinone derivative [1]. Approved by the U.S. Food and Drug Administration (FDA) in 2000, it was the first antibiotic of its class authorized for human use [2]. Linezolid has demonstrated antibacterial activity against numerous pathogens, including *Mycobacteria, Nocardia, Corynebacterium,* and anaerobes. In addition, in vitro studies have reported inhibitory effects against certain fungi [3]. Its antimicrobial activity results from inhibition of bacterial protein synthesis via binding to ribosomal RNA within the 30S and 50S subunits [4].

Currently, linezolid is approved by the FDA for the treatment of uncomplicated and complicated skin infections, community-acquired pneumonia, nosocomial pneumonia, and infections caused by Staphylococcus aureus resistant to penicillin-class antibiotics, cephalosporins, vancomycin, and methicillin, as well as vancomycin-resistant enterococcal infections [1,2]. Antibiotic therapy for bone and joint infections commonly requires treatment durations of six weeks or longer. Although linezolid is considered an effective option for these infections, its use beyond 28 days may cause severe adverse effects [5], with the risk increasing with treatment duration. Reported side effects include thrombocytopenia, peripheral neuropathy, lactic acidosis, and optic neuropathy [6]. Although generally well tolerated, prolonged administration has been linked to lactic acidosis and hepatic injury [7]. Following linezolid exposure, increases in hepatocyte damage markers and oxidant parameters, along with decreases in antioxidant levels, have been reported [8]. This adverse effect is attributed to disturbances in mitochondrial oxidative phosphorylation [9]. Linezolid-associated lactic acidosis and liver failure may have serious consequences that require treatment discontinuation [10].

TPP, the active metabolite of thiamine (vitamin B1), was investigated in this study for its potential protective effects against linezolid-induced toxicity [11]. Thiamine is present in meat, grains, legumes, and nuts [12] and is converted to its active form, TPP, by the enzyme thiamine pyrophosphokinase [13]. As a cofactor of pyruvate dehydrogenase, TPP participates in the conversion of pyruvate to acetyl-CoA. Inadequate TPP leads to pyruvate being diverted to lactate, promoting lactic acid accumulation and the subsequent development of lactic acidosis [14,15]. Lactate production in humans arises from the conversion of intracellular alanine and glucose to pyruvate via lactate dehydrogenase (LDH) [16,17]. Previous studies have shown that TPP prevents lactate accumulation, improves glucose metabolism, and enhances aerobic capacity [11]. These findings suggest that TPP may have therapeutic potential in managing linezolid-induced lactic acidosis and hepatic injury. Given that thiamine is the metabolic precursor of TPP, we hypothesized that while TPP would directly counteract mitochondrial dysfunction induced by linezolid, thiamine may exhibit a less pronounced or no protective effect unless effectively converted to TPP intracellularly. However, no previous studies have investigated the effects of TPP on linezolid-induced experimental liver damage and lactic acidosis. Therefore, this study aimed to evaluate the protective effects of TPP against linezolid-induced liver damage and lactic acidosis in rats.

## 2. Materials and Methods

### 2.1. Experimental Animals

A total of 24 male Wistar albino rats (260–278 g) were used in the study. All rats were provided by the Erzincan Binali Yildirim University Experimental Animals Application and Research Center. The rats were housed in groups (*n* = 6) under appropriate laboratory conditions at a room temperature of 22 ± 2 °C with a 12 h light/dark cycle. The rats were fed with animal feed and normal tap water before and during the experiment. Experimental methods were approved by the local Animal Experimentation Ethics Committee of Erzincan Binali Yildirim University (Date: 27 March 2025, meeting/decision no: 03/12).

### 2.2. Chemicals

Thiamine and thiamine pyrophosphate (cocarboxylase hydrochloride) were obtained from Biofarma (Moscow, Russia). The purity of TPP is 98% or higher, as stated by the manufacturer (Biofarma, Moscow, Russia). Linezolid was obtained from Pfizer (Istanbul, Turkey). The thiopental sodium was obtained from IE Ulagay (Istanbul, Turkey).

### 2.3. Experimental Groups

The rats were distributed into four distinct groups: healthy group (HG), linezolid (LZD), thiamine + linezolid (TLZD), and TPP + linezolid (TPLZD).

### 2.4. Experimental Procedure

In the TLZD (*n* = 6) group, thiamine (20 mg/kg) was injected intraperitoneally (ip), while the TPLZD (*n* = 6) group received TPP (20 mg/kg) via the same route. The doses of thiamine and thiamine pyrophosphate (20 mg/kg) used in this study were selected based on previous experimental studies investigating oxidative stress and drug-induced organ injury, in which these doses were found to be effective [18]. Both thiamine and TPP were dissolved in sterile isotonic saline (0.9% NaCl) prior to administration. The HG (healthy group) and LZD (*n* = 6) groups administered physiological saline solution (0.9% NaCl). One hour after the administration of thiamine, TPP, or 0.9% NaCl, linezolid (125 mg/kg) was orally administered to the LZD, TLZD, and TPLZD groups. Linezolid was given twice daily (every 12 h, a total of 250 mg/kg/gün) [19], while thiamine and TPP were administered once daily for four weeks. After the experimental process, all rats were euthanized with thiopental sodium (50 mg/kg, ip) [20], and liver tissues were collected. Levels of malondialdehyde (MDA), total glutathione (tGSH), superoxide dismutase (SOD), and catalase (CAT) were measured in the liver tissues. Furthermore, liver tissues were subjected to histopathological evaluation. Prior to euthanasia, blood was collected from the tail vein and analyzed for LDH, alanine aminotransferase (ALT), aspartate aminotransferase (AST), and TPP.

### 2.5. Biochemical Analysis

#### 2.5.1. Determination of MDA, GSH Levels, SOD and CAT Activities in Liver Tissue

The quantification of MDA and GSH levels in the tissues was conducted in accordance with the manufacturer’s instructions, employing ELISA kits from ELK Biotechnology CO., Ltd. (catalog numbers, respectively: ELK8612, ELK8577, Wuhan, China). The assessment of SOD and CAT activity was conducted utilizing kits from ELK Biotechnology CO., Ltd. (catalog numbers, respectively: ELK8178, ELK5986, Wuhan, China).

#### 2.5.2. Determination of Lactate Levels

Blood samples from the rats were collected using lithium heparin-coated syringes. The measurement of lactate levels was conducted by means of the ABL800 FLEX blood gas analyzer (Radiometer; Copenhagen, Denmark), based on the fluorescence optical electrode method.

#### 2.5.3. Determination of LDH Activities

The quantitative analysis of serum LDH (P-L) was performed using a spectrophotometric method on a Roche brand Cobas 8000 autoanalyzer (Roche Diagnostics GmbH, Mannheim, Germany). LDH catalyzes the conversion of pyruvate to L-lactate and NAD+ from pyruvate and NADH. Pyruvate + NADH + H^+^→(LDH) L-lactate + NAD^+^. The catalytic LDH activity is directly related to the initial rate of NADH oxidation. The reduction in wavelength (340 nm) was measured, thus allowing the calculation of the extinction coefficient.

#### 2.5.4. Determination of ALT and AST Activities

Venous blood samples were gathered into tubes without the addition of an anticoagulant agent. Following clotting, the serum was isolated by centrifugation and preserved at −80 °C until analysis. The Cobas 8000 autoanalyzer (Roche Diagnostics GmBH, Mannheim, Germany) with kits (Roche Diagnostics, GmBH, Mannheim, Germany) was utilized to measure serum AST and ALT activities spectrophotometrically.

#### 2.5.5. Determination of TPP Levels

To determine serum TPP levels, blood samples were treated with an equal volume (1:1) of 10% trichloroacetic acid (TCA) to precipitate proteins and extract TPP. The samples were vortexed for 5 min and subsequently centrifuged at 5000 rpm for 10 min. The resulting supernatant was reacted in an alkaline medium containing potassium ferricyanide (K_3_[Fe(CN)_6_]) and 20% sodium hydroxide to form fluorescent thiochrome derivatives. The reaction mixture was then injected into a high-performance liquid chromatography column for separation. In the TPP determination, a fluorescence detector (Agilent Technologies, Waldbronn, Germany) with an excitation wavelength of 375 nm and an emission wavelength of 435 nm was utilized. The chromatographic separation was performed using a mobile phase consisting of 74% KH_2_PO_4_ buffer (pH 6.2) and 26% methanol. TPP peaks were eluted at approximately 2 and 8 min.

### 2.6. Histopathological Method

After the necropsy of the rats, liver tissues were collected and fixed in 10% neutral formalin solution. The tissues were then dehydrated with graded alcohol, cleared in xylol, and embedded in paraffin blocks according to standard histological procedures. Four-micrometer-thick sections of tissue were cut and mounted on slides. The slides were then stained with hematoxylin and eosin (H&E), and a semiquantitative evaluation was performed to assess the degree of hydropic degeneration and necrosis in hepatocytes, as well as the extent of mononuclear cell infiltration in the periportal areas. The severity of these changes was assigned a numerical value: none (0), mild (1), moderate (2), and severe (3). The pathologist performing the histological evaluations was blinded to the group assignments during analysis.

### 2.7. Statistical Analysis

Statistical analyses were conducted using the “SPSS for Windows, version 22.0” statistical software. The data were expressed as “mean ± standard deviation (X ± SD)”. The Shapiro–Wilk Test showed that biochemical data are normally distributed. Therefore, one-way ANOVA was used for analysis. The homogeneity of variances was assessed using the Levene test. Based on the outcome of this test, post hoc comparisons were performed using the Tukey test when variances were homogeneous, and the Games–Howell test when variances were not homogeneous. The Mann–Whitney U test was used in the analysis of histopathological semiquantitative data. *p* values below 0.05 were considered statistically significant.

## 3. Results

### 3.1. Biochemical Results

#### 3.1.1. MDA and tGSH Level Analysis Results

**Figure 1:**
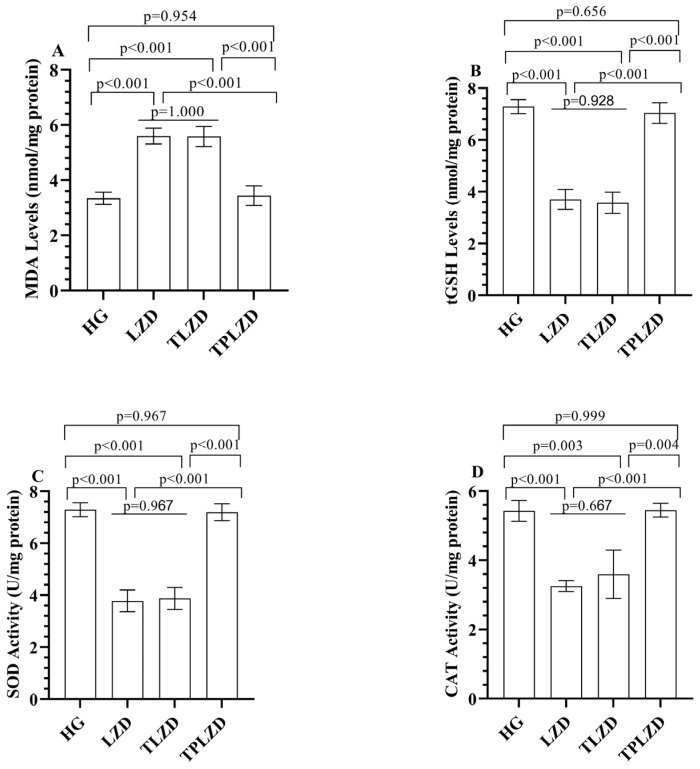
(**A**) MDA; (**B**) tGSH; (**C**) SOD; and (**D**) CAT levels in liver tissues of experimental groups. Bars indicate mean ± standard deviation, *n* = 6 (per group). p < 0.05 was determined as statistical significance. One-way ANOVA followed by Tukey’s post hoc test was used for statistical analysis. Exact p-values above the bars represent significant pairwise comparisons among HG, LZD, TLZD, and TPLZD groups. Providing exact *p*-values enhances interpretability and ensures transparency in statistical reporting, especially when multiple comparisons are involved. MDA, malondialdehyde; tGSH, total glutathione; SOD, superoxide dismutase; CAT, catalase; HG, healthy group; LZD, linezolid group; TLZD, thiamine + linezolid group; TPLZD, TPP + linezolid group.

As illustrated in Figure 1A,B, compared to HG, LZD treatment increased hepatic MDA levels by approximately 92% (from 3.6 ± 0.3 to 6.9 ± 0.4 nmol/mg protein). In the TLZD, MDA levels remained elevated (6.8 ± 0.3), whereas TPLZD markedly reduced MDA levels to 4.7 ± 0.3, approaching control values. Conversely, tGSH levels were reduced by approximately 51% in the LZD group (from 8.3 ± 0.4 to 4.1 ± 0.2 nmol/mg protein). Thiamine had minimal effect (4.3 ± 0.3), while TPP restored tGSH levels to 6.5 ± 0.3.

#### 3.1.2. SOD and CAT Analysis Results

As depicted in Figure 1C,D, linezolid decreased hepatic SOD activity by nearly 51% (from 7.7 ± 0.3 to 3.8 ± 0.3 U/mg protein). SOD activity remained suppressed in the TLZD group (4.0 ± 0.2), while it improved significantly in the TPLZD group (7.3 ± 0.3). CAT activity dropped by 44.6% following LZD treatment (from 5.6 ± 0.3 to 3.1 ± 0.3 U/mg protein). This was only slightly improved in the TLZD group (3.2 ± 0.2) but was nearly normalized in the TPLZD group (5.1 ± 0.3).

#### 3.1.3. Lactate Level Analysis Results

**Figure 2:**
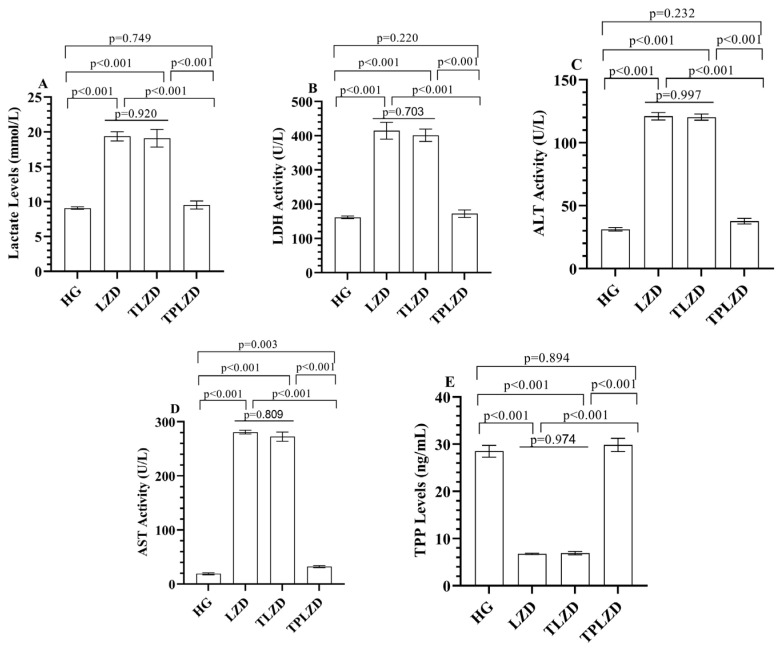
(**A**) Lactate; (**B**) LDH; (**C**) ALT; (**D**) AST; and (**E**) TPP levels in serum of experimental groups. Bars indicate mean ± standard deviation, *n* = 6 (per group). *p* < 0.05 was determined as statistical significance. One-way ANOVA followed by Tukey’s post hoc test was used for statistical analysis. Exact *p*-values above the bars represent significant pairwise comparisons among HG, LZD, TLZD, and TPLZD groups. Providing exact p-values enhances interpretability and ensures transparency in statistical reporting, especially when multiple comparisons are involved. LDH, lactate dehydrogenase; ALT, alanine aminotransferase; AST, aspartate aminotransferase; TPP, thiamine pyrophosphate; HG, healthy group; LZD, linezolid group; TLZD, thiamine + linezolid group; TPLZD, TPP + linezolid group.

As illustrated in Figure 2A, serum lactate concentrations increased approximately threefold after linezolid administration (from 6.2 ± 0.4 to 19.1 ± 1.0 mmol/L). Thiamine had a negligible effect (18.8 ± 0.9), whereas TPP reduced lactate to 8.9 ± 0.5 mmol/L—close to control levels.

#### 3.1.4. LDH Activity Analysis Results

As demonstrated in Figure 2B, LDH activity increased from 218.6 ± 9.5 to 451.8 ± 12.4 U/L following LZD exposure (a 107% increase). The TLZD group showed no improvement (439.1 ± 13.1), while the TPLZD group exhibited a significant reduction to 228.5 ± 10.2 U/L.

#### 3.1.5. ALT and AST Activities Analysis Results

As shown in Figure 2C,D, LZD elevated serum ALT and AST levels from 52.3 ± 3.7 to 139.4 ± 6.2 U/L and from 76.8 ± 4.1 to 274.6 ± 11.3 U/L, respectively. These represent increases of 166% and 257%. Thiamine did not significantly reduce these values (ALT: 136.7 ± 7.3; AST: 262.4 ± 10.1), whereas TPP treatment notably reduced ALT to 83.7 ± 5.1 and AST to 171.5 ± 8.4 U/L.

#### 3.1.6. TPP Level Analysis Results

As shown in Figure 2E, serum TPP levels decreased from 31.4 ± 1.1 to 12.3 ± 0.7 ng/mL in the LZD group (a > 60% reduction). Thiamine supplementation did not restore TPP levels (12.4 ± 0.6), whereas direct TPP administration normalized them to 28.5 ± 0.9 ng/mL.

### 3.2. Histopathological Results

**Figure 3:**
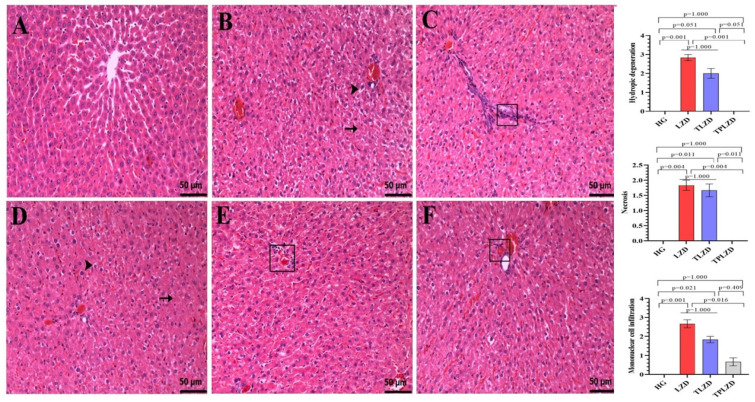
Histopathological appearance of liver tissues belonging to the (**A**) HG; (**B**,**C**) LZD; (**D**) TLZD; and (**E**,**F**) TPLZD groups (H&E). (**A**) HG group. Normal histological appearance in liver tissue. (**B**) LZD group. Severe hydropic degeneration (arrowhead) and moderate necrosis (arrow) in liver tissue. (**C**) LZD group. Severe mononuclear cell infiltration (square) in the periportal areas of the liver tissue. (**D**) TLZD group. Moderate hydropic degeneration (arrowhead) and moderate necrosis (arrow) appearance in liver tissue. (**E**) TLZD group. Appearance of moderate mononuclear cell infiltration (square) in the periportal areas of the liver tissue. (**F**) TPLZD group. Appearance of mild mononuclear cell infiltration (square) in the periportal areas of the liver tissue. Bars indicate mean ± standard deviation, *n* = 6 (per group). The Mann–Whitney U test was used in the analysis of histopathological semiquantitative data. *p* < 0.05 was determined as statistical significance. HG, healthy group; LZD, linezolid group; TLZD, thiamine + linezolid group; TPLZD, TPP + linezolid group. Microscopic magnification: 20×.

Histopathological analyses demonstrated statistically significant differences among the groups (Figure 3). The liver tissues of the HG rats exhibited a normal histological morphology (Figure 3A). In the LZD group, severe hydropic degeneration and moderate necrosis were seen in hepatocytes (Figure 3B). Severe mononuclear cell infiltrations were detected in the periportal areas in this group (Figure 3C). In the TLZD group, moderate hydropic degeneration and moderate necrosis were also seen in hepatocytes (Figure 3D). Additionally, moderate mononuclear cell infiltrations were observed in the periportal areas in this group (Figure 3E). In the TPLZD group, no hydropic degeneration and necrosis were observed in hepatocytes, but mild mononuclear cell infiltrations were observed in the periportal areas (Figure 3F).

## 4. Discussion

In this study, the potential protective effects of thiamine and TPP against possible liver injury and lactic acidosis induced by linezolid were investigated in rats. Our findings showed that linezolid significantly increased MDA levels in liver tissue, as well as LDH, ALT, AST and lactate levels in the blood, while it decreased tissue levels of tGSH, SOD, CAT, and TPP. MDA is considered a byproduct of lipid peroxidation, whereas tGSH, SOD, and CAT are known as endogenous antioxidants [21].

In the extant literature, an increase in oxidants and a decrease in antioxidants are considered to be indicative of oxidative stress [22]. In a study that is consistent with our experimental results, an increase in oxidant and hepatocyte damage markers and a decrease in antioxidants were observed after linezolid treatment [8]. Moreover, substantial evidence indicates that linezolid toxicity is associated with enhanced production of free oxygen radicals and a concomitant suppression of the antioxidant defense mechanisms [7,8,23]. In this process, the accumulation of lipid peroxidation products such as MDA and membrane damage are particularly noteworthy [24]. It is therefore evident that MDA is an important marker used to measure levels of oxidative stress in tissues [25]. In our study, the increase in oxidant levels and decrease in antioxidant levels observed in linezolid-treated animals are consistent with previous reports in the literature.

There are various experimental and preclinical studies in the literature that demonstrate the potential protective effects of antioxidant agents against linezolid-induced hepatotoxicity. tGSH, SOD, and CAT are important endogenous antioxidant mechanisms that protect cells against oxidative stress-induced damage [26]. Azzam and colleagues reported that linezolid caused hepatotoxic effects in rats by increasing oxidant levels and decreasing antioxidant levels such as GSH and CAT [27]. Similarly, Vivekanandan and colleagues demonstrated that linezolid-induced hepatotoxicity was associated with antioxidant suppression [24]. The findings of the current study are consistent with these previous reports, demonstrating a significant reduction in tGSH, SOD, and CAT key components of the antioxidant defense system in the group of animals treated with linezolid.

To evaluate linezolid-induced liver injury, serum levels of LDH, ALT, and AST activities were analyzed. Elevated serum levels of these enzymes are considered to reflect hepatic cellular damage [28]. Our experimental findings indicated that serum LDH, ALT, and AST activities were significantly elevated in the LZD rats. LDH is an important enzyme of the anaerobic metabolic pathway found in almost all body tissues, and its quantification is clinically important because the serum concentration of LDH isozymes reflects tissue-specific pathological conditions [29]. Although LDH is not a liver-specific marker, it has been utilized in both preclinical and clinical studies to assess hepatic injury [30,31,32]. Moreover, in humans, lactate production occurs through the conversion of intracellular alanine and glucose to pyruvate via the enzyme LDH [16,17]. ALT and AST are metabolic enzymes, and their increased concentrations in the blood are indicative of hepatocellular damage [31]. These two enzymes, particularly more specific to the liver, have been widely used as biomarkers in numerous studies investigating drug-induced liver pathologies [7,33,34]. These previous studies corroborate and align with the outcomes of our research.

Linezolid use can lead to serious complications such as lactic acidosis, resulting in life-threatening multiple organ failure [35]. The existence of this serious condition caused by linezolid has been demonstrated in numerous case reports in the literature [35,36,37]. In these publications, renal replacement therapy has been applied to patients with severe linezolid-induced lactic acidosis to improve acidosis. In addition, it has been pointed out that risk factors such as thiamine deficiency should be corrected [38]. Thiamine supplementation has also been used to reverse adverse events associated with linezolid [39].

Based on the information available in the literature, the potential preventive impact of thiamine against oxidative liver injury and lactic acidosis induced by linezolid was evaluated in treated animals. However, contrary to the findings reported in the literature, no significant changes were observed in oxidative stress and hyperlactatemia parameters in the group treated with thiamine. This finding suggests that TPP, the active form of thiamine, may offer more effective results in regulating mitochondrial function associated with linezolid-induced toxicity. TPP acts as a cofactor for pyruvate dehydrogenase (PDH), which is essential in the conversion of pyruvate to acetyl-CoA [14]. In the presence of TPP deficiency or PDH inhibition, pyruvate is instead shunted toward lactate formation via lactate dehydrogenase, leading to lactic acidosis [15]—a known complication of linezolid due to its mitochondrial toxicity [9]. Previous studies in the literature have demonstrated that TPP exhibits greater protective efficacy than thiamine in drug-induced oxidative liver injury [18,40]. In line with the existing literature, our study also revealed a marked improvement in biochemical parameters in the group treated with TPP. Our results indicate that TPP restored antioxidant enzyme activities (tGSH, SOD, CAT) and reduced MDA levels. This suggests that TPP counteracts the mitochondrial dysfunction and reactive oxygen species (ROS) overproduction induced by linezolid, consistent with previously reported antioxidant properties of TPP [40,41]. A comparable recovery in LDH, ALT, and AST levels was observed, with values nearing those of the healthy controls.

Our study demonstrated a significant reduction in thiamine pyrophosphate (TPP) levels in the linezolid (LZD) group. Similarly, animals treated with thiamine also exhibited reduced TPP concentrations. However, in animals directly treated with TPP, linezolid did not significantly alter circulating TPP levels. These findings suggest that linezolid may interfere with thiamine metabolism. The observed decrease in TPP levels in the LZD group implies a possible inhibition of thiamine pyrophosphokinase (TPK), the enzyme responsible for converting thiamine to its active form, TPP. This hypothesis aligns with previous reports indicating that certain drugs can disrupt thiamine metabolism [13]. While we did not directly measure enzyme activity, this hypothesis warrants further investigation in future studies.

Moreover, the literature highlights the antioxidant properties of TPP and its role in preventing hyperglycemia [4]. Our experimental findings support these previously reported effects of TPP [21]. Prior studies have shown that TPP, the active metabolite of thiamine, prevents lactate accumulation, improves glucose metabolism, and enhances aerobic capacity [11].

Our histopathological findings support the biochemical results, revealing widespread hydropic degeneration and necrosis in hepatocytes, along with severe mononuclear cell infiltration in the periportal area of the linezolid-treated group. In line with our observations, previous studies in the literature have demonstrated that linezolid induces hepatotoxicity by inhibiting mitochondrial protein synthesis and through its degenerative effects, as evidenced by histopathological analyses [24]. Moreover, no significant histopathological improvement was observed in the thiamine group, whereas in the TPP group, neither hepatocellular degeneration nor necrosis was detected. Only mild mononuclear cell infiltration was observed in the periportal region, suggesting that TPP provided significant protection against linezolid-induced hepatic injury. Histopathological data from previous studies that have documented the hepatoprotective effects of TPP further confirm our results and suggest that TPP may be a promising treatment option [21].

Several limitations of this study should be acknowledged. First, the dose of linezolid used in our experimental protocol (250 mg/kg/day) is substantially higher than that typically administered in clinical settings. This elevated dose was intentionally selected to reliably induce measurable hepatic injury and lactic acidosis within a relatively short experimental timeframe [19]. Nevertheless, this discrepancy limits the direct translatability of the findings to human clinical scenarios.

Second, the study did not include groups treated with thiamine or TPP alone. While the primary objective was to assess the protective effects of these compounds against linezolid-induced toxicity, the absence of standalone thiamine and TPP groups precludes a precise evaluation of their individual baseline effects. Including these groups in future research would enhance the mechanistic understanding of each compound’s pharmacodynamic profile.

Finally, it remains unclear why linezolid markedly reduced circulating TPP levels, why thiamine failed to exert a protective effect, and why TPP was effective in mitigating toxicity. One plausible explanation is that linezolid may impair the activity of thiamine pyrophosphokinase, the enzyme responsible for converting thiamine to its active form, TPP. Further studies are warranted to directly investigate whether linezolid interferes with this enzymatic conversion pathway.

## 5. Conclusions

In conclusion, the data obtained showed that linezolid created an oxidative stress environment, which caused hepatocellular damage biochemically and histopathologically. In addition, the increased levels of liver function test markers in the blood confirmed the functional liver damage potential of linezolid. It was also observed that linezolid caused hyperlactatemia. Consequently, our findings clearly demonstrate the hepatotoxic effects of linezolid, both in terms of oxidative damage markers and metabolic enzyme levels. While TPP treatment significantly reduced these effects, the protective efficacy of thiamine appeared to be limited. Based on these results, TPP supplementation may warrant further investigation as a protective strategy in preclinical models of drug-induced mitochondrial toxicity, such as that caused by linezolid.

## Data Availability

Data from the research can be obtained from the corresponding author upon reasonable request.

## Abbreviations

The following abbreviations are used in this manuscript:

TPP	Thiamine Pyrophosphate
MDA	Malondialdehyde
tGSH	Total Glutathione
SOD	Superoxide Dismutase
CAT	Catalase
LDH	Lactate Dehydrogenase
ALT	Alanine Aminotransferase
AST	Aspartate Aminotransferase
FDA	Food and Drug Administration
